# Stretchable Water-Free Liquid Crystalline Polyelectrolyte Complexes

**DOI:** 10.1021/acsapm.6c01534

**Published:** 2026-08-04

**Authors:** Xiaohong Liu, Yuxuan Zhang, Xixi Wu, Rui Li, Daniele Parisi, Giuseppe Portale, Marleen Kamperman, Julien Es Sayed

**Affiliations:** † Polymer Science Group, Zernike Institute for Advanced Materials, Faculty of Science and Engineering, 332143 University of Groningen, Nijenborgh 3, Groningen 9747 AG, The Netherlands; ‡ KINGFA Sci. & Tech. Co. Ltd., No. 33, Kefeng Road, Science Town, Guangzhou 510663, China; § Physical Chemistry of Polymeric and Nanostructured Materials, Zernike Institute for Advanced Materials, Faculty of Science and Engineering, 332143 University of Groningen, Nijenborgh 3, Groningen 9747 AG, The Netherlands; ∥ School of Medicine & Nursing, Huzhou University, No. 759, Erhuan East Road, Huzhou 313000, China; ⊥ Department of Chemical Engineering, Engineering and Technology Institute Groningen, 84790 University of Groningen, Nijenborgh 3, Groningen 9747 AG, The Netherlands; # Biotechnology Centre, 3647 The Silesian University of Technology, B. Krzywoustego 8, Gliwice 44-100, Poland

**Keywords:** polyelectrolyte complex, charge density, thermal actuator, liquid crystalline elastomer, molecular alignment

## Abstract

Polyelectrolyte complexes (PECs), formed by electrostatic association of oppositely charged polyelectrolytes, are promising low-environmental-impact materials. However, their extreme brittleness in the absence of water and a general lack of stimuli-responsive functionality severely limit practical applications. Here, we introduce a strategy to overcome both limitations by designing liquid crystalline polyelectrolyte complexes (LCPECs). Main-chain liquid crystalline (LC) polyanions with systematically varied charge densities and neutral flexible domains are first synthesized via a facile one-pot two-step polymerization combining Michael addition and thiol–ene chemistry. The LC polyanions are then complexed with poly­(diallyldimethylammonium chloride) (PDADMAC) to form LCPECs with tunable properties. X-ray scattering reveals a fractal-like network with nanoscale phase separation between electrostatically associated and neutral flexible domains, alongside nematic ordering of the mesogens. Increasing the neutral monomer content lowers the glass transition temperature from 58 °C to −10 °C and transforms the mechanical response from stiff and brittle to soft and highly stretchable, achieving an elongation of 1160%, far exceeding the stretchability of any previously reported water- and salt-free PEC. Rheological analysis shows that higher charge density raises both moduli and delays chain relaxation, confirming that electrostatic interactions function as tunable physical cross-links. Uniaxial stretching induces macroscopic mesogen alignment, enabling reversible thermal actuation with 20% dimensional change upon cycling across the LC–isotropic transition. Furthermore, well-defined mesh structures are successfully prepared by melt electrowriting, demonstrating the processability of these materials through advanced additive manufacturing. This work establishes tuning of ionic cross-link density and backbone flexibility as a general strategy for transforming inherently brittle PECs into mechanically robust, stimuli-responsive, and processable materials.

## Graphical abstract

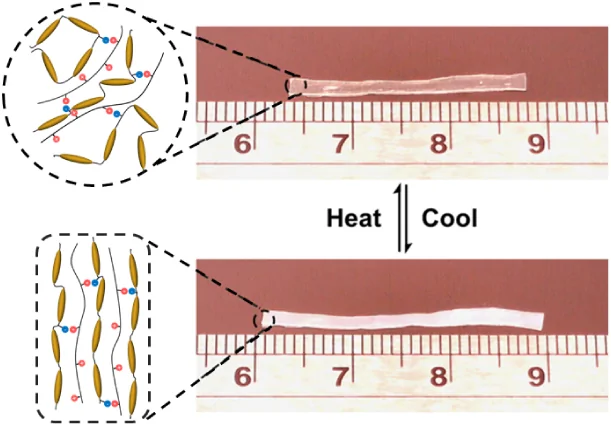

## 1 Introduction

Polyelectrolyte complexes (PECs) are a class of polymer materials formed by the electrostatic association of oppositely charged macromolecules.^1^ PECs have attracted great interest because they can be processed into tough functional materials, such as filtration membranes, coatings, and load-bearing fibers, from homogeneous aqueous mixtures of their constituent polymers and salt.^1–6^ Solidification of the liquid dope is commonly achieved by desalting, which reveals the electrostatic interactions originally screened by the excess salt. Over the past decade, significant progress has been made in understanding the relationships between processing routes, chemical composition, and structure–property relationships, enabling a more rational design of PECs. Indeed, polyelectrolytes with tunable charge densities, nonelectrostatic supramolecular stickers, and variable main-chain rigidities have been designed to develop PECs with tailored mechanical properties.^7–13^ Despite this progress, two major limitations still hinder the widespread adoption of PECs as replacements for commonly used functional polymer materials. The first limitation is that their processability and mechanical properties are fundamentally tied to their degree of hydration. They become extremely brittle upon dehydration due to strong Coulombic interactions, severely limiting their use in nonaqueous environments.^14–21^ Recently, van Lange et al. proposed an innovative PEC system that exhibits unusual rubber-like properties under water-free conditions.^22–25^ The so-called “compleximers” are processed by complexing specifically designed polyelectrolytes in which bulky, hydrophobic spacer groups are covalently grafted to the charge-carrying moieties. These spacer groups plasticize the material, by separating the electrostatically associating units. Nevertheless, this approach relies heavily on side-chain modification, and their stretchability remains marginal compared to conventional elastomers, even in the presence of ionic liquids used as external plasticizers.

The second limitation of current PEC materials is that they are essentially static, lacking stimuli-responsive capabilities. They miss the fourth-dimensional parameter through which properties and shape can be temporally controlled.^26,27^ The fourth dimension is commonly described as responsivity to external stimuli, which can be encoded and programmed into a material’s chemical composition and/or microarchitecture to induce autonomous transformations in shape, size, or mechanical properties. Such actuators are gaining increasing interest in the fields of soft robotics and 4D printing. Liquid crystalline elastomers (LCEs) represent a class of anisotropic polymer materials that exhibit precisely these actuation properties, typically arising from the disruption and restoration of mesogen alignment or from directional swelling and deswelling.^28–30^ A common strategy for preparing well-aligned LCEs involves mechanical deformation of a partially cross-linked LCE followed by completion of network formation through a second polymerization step. Although a certain cross-linking density is essential for reversible actuation, covalent cross-links hinder material recycling and reprogramming.^31^ Recently, LCEs incorporating dynamic cross-links, including dynamic covalent bonds and hydrogen bonds, have been explored, as they not only enable reprocessability but also simplify the programming by eliminating the second polymerization step.^32–34^ In this context, electrostatic interactions, which are inherently reversible and tunable, represent an unexplored yet conceptually appealing type of dynamic cross-link for liquid crystalline actuator systems.

Herein, we report a novel PEC system that simultaneously addresses the brittleness and lack of responsiveness of conventional water- and salt-free PECs by balancing the density of ionic cross-linking and flexible segments and integrating LC functionality. Liquid crystalline polyelectrolyte complexes (LCPECs) were prepared through a two-step synthetic approach, in which LC polyanions with systematically varied charge densities were first synthesized via sequential Michael addition and thiol–ene reactions, followed by complexation with cationic poly­(diallyldimethylammonium chloride) (PDADMAC). X-ray scattering indicated a fractal-like structure and nanoscale phase separation between the ion pairs and neutral segments, alongside a nematic phase in the LCPECs. With decreasing charge density and increasing neutral segments, chain mobility was improved, resulting in a lower glass transition temperature (*T*_g_) and a gradual change of mechanical behavior from brittle to stretchable. Mechanical stretching induced mesogen alignment, which enabled thermally driven reversible actuation around the LC–isotropic transition temperature. Finally, we showcased the processability of these materials through the melt electrowriting of well-defined mesh structures.

## 2 Experimental Section

### 2.1 Materials

1,4-Bis­[4-(3-acryloyloxypropyloxy)­benzoyloxy]-2-methylbenzene (98%, monomer A) was purchased from BLDpharm. Taurine (monomer B_1_), sodium hydroxide (98%), poly­(diallyldimethylammonium chloride) (PDADMAC, average Mw 400,000–500,000, 20 wt % in H_2_O), and 2,2′-(ethylenedioxy)­diethanethiol (monomer B_2_, 95%) were purchased from Sigma-Aldrich. Note that PDADMAC was freeze-dried before use. 1,8-Diazabicyclo[5.4.0]­undec-7-ene (DBU, 98%), diethylene glycol dimethyl ether (diglyme, 99%), and dimethyl sulfoxide (DMSO) were purchased from TCI.

### 2.2 Characterization

^1^H NMR experiments were performed on a Bruker Avance III HD spectrometer operating at 400 MHz. Polarized optical microscopy (POM) images were taken with a DM2700p from Leica. Uniaxial tensile tests were performed with an Instron 24SC-1kN from Instron, USA. Melt electrowriting was conducted with a MEW device (Spraybase A-1204–0001–01D, Ireland). Thermogravimetric analysis (TGA) experiments were performed using a TGA Discovery series 5500 instrument. Differential scanning calorimetry (DSC) experiments were performed using a DSC Q1000, TA Instruments. For the TGA measurements, the polyelectrolyte complexes were heated to 700 °C at 10 °C/min in air. For DSC measurements, the polyelectrolyte complexes were cooled down from 150 °C to −50 °C and heated back up to 150 °C at 10 °C/min. The ramp cycles were repeated three times for each sample, and the second cycle was used for analysis. SAXS and WAXS experiments were performed at the Multipurpose X-ray Instrument for Nanostructural Characterization (MINA) at the University of Groningen. The instrument is equipped with a high-intensity Cu rotating anode X-ray source, providing a parallel collimated X-ray beam with a photon wavelength of λ = 0.1543 nm. The scattering patterns were collected using a Bruker Vantec2000 detector (pixel size of 68 μm × 68 μm) and a Bruker Vantec500 detector (pixel size of 136 μm × 136 μm). Rheological measurements were performed on an Anton Paar MCR302e strain-controlled instrument.

### 2.3 Synthesis

Monomer B_1_ (1302 mg, 10.2 mmol for S_1_; 651 mg, 5.1 mmol for S_2_; and 319 mg, 2.5 mmol for S_3_) dissolved in NaOH aqueous solution (102 mg, 2.5 mmol, in 3.19 mL of water) and monomer A dissolved in diglyme (6 g, 10.2 mmol for all samples, in 24 mL of diglyme) were added to a round-bottom flask. The mixture was stirred at 60 °C overnight before being cooled to room temperature. Monomer B_2_ (0.92 g, 5.09 mmol for S_2_, and 1.39 g, 7.65 mmol for S_3_) and 5 drops of DBU were added, and the mixture was further stirred at room temperature for 6 h. After the two-step polymerization, DMSO (half of the volume of diglyme) was added to the mixture and heated to 50 °C to obtain a completely homogeneous solution. PDADMAC (1680 mg, 10.2 mmol for S_1_; 840 mg, 5.1 mmol for S_2_; and 412 mg, 2.5 mmol for S_3_) was dissolved in deionized water (4.12 mL) and added dropwise with rigorous stirring. The mixture was further stirred at room temperature overnight, and the LCPEC was washed with deionized water 3 times and dried in a vacuum oven. For sample S_4_, monomers A (2 g, 3.4 mmol) and B_2_ (619 mg, 3.4 mmol) were dissolved in diglyme (8 mL) and a drop of DBU was added. The mixture was agitated at room temperature overnight and precipitated in EtOH. The samples were hot-pressed at 100 °C and 50 kPa for 5 min into 0.5 mm thick pies before use.

### 2.4 Small-Angle (SAXS) and Wide-Angle (WAXS) X-ray Scattering

The samples were loaded in a copper sample holder with a thickness of 1 mm and sealed with Kapton tapes. The SAXS data were acquired using two different sample-to-detector distances of 3 and 0.24 m, and the data were merged for the SAXS curves after subtracting the background. The WAXS measurements were performed using a sample-to-detector distance of 0.08 m. The sample-to-detector distance and the beam center position were calibrated by using the scattered rings from a standard silver behenate powder sample. The measurements were performed at 20 °C. The SAXS and WAXS patterns were converted into 1D scattering intensity profiles by using Fit2D software.

### 2.5 Mechanical Characterizations

The samples were hot-pressed at 100 °C under 50 kPa pressure for 5 min into 0.5 mm thick and 15 mm diameter cylinders. The specimens were loaded on a rheometer preheated to 150 °C. A 10 mm parallel plate geometry was used for all measurements except for S_4_ (measured with a 25 mm parallel plate), and the excess material was trimmed. The samples were measured every 1 °C at 1 °C/min down to 20 °C. For tensile testing, the samples were hot-pressed at 100 °C under 50 kPa pressure for 5 min into 0.5 mm thick dog-bone specimens. The total length was 2.5 cm, and the neck was 1 cm in length and 2.4 mm in width. The specimens were stretched at 0.5 mm/s (strain rate of 0.05 s^–1^) at room temperature. Before mechanical testing, all the samples were equilibrated at 20 °C (room temperature) under RH = 50% relative humidity unless otherwise stated.

### 2.6 Alignment of the Specimens and Thermal Actuation

S_3_ was used for mechanical alignment and thermal actuation investigation, and the corresponding dog-bone specimens were prepared as stated above. The specimens were stretched to 500% strain (assuming only the neck was stretched) and kept in an oven preheated to 100 °C for a minute. Then, the specimens were cooled down to room temperature while being strained. Finally, the specimens were heated to 90 °C and cooled down to room temperature to remove any residual stress. A preliminary experiment revealed that the actuation took place mainly between 50 and 90 °C. The aligned specimen was put on a hot plate preheated to 50 °C, and the thermal-driven actuation was investigated in a heating–cooling cycle between 90 and 50 °C.

### 2.7 Melt Electrowriting

S_3_ was printed following a previously published procedure.^35^ The LCPECs were melted at 150 °C in the stainless-steel syringe cartridge equipped with a 0.15 mm nozzle for an hour. 3-layer structure meshes with a strand diameter of 50 and 100 μm interfiber distance were printed. A voltage of 3.5 kV and a pressure of 0.5 bar were used. The printing distance between the nozzle and the collector was kept at 151.5 mm and the collecting velocity was 10 mm/s.

## 3 Results and Discussion

### 3.1 Preparation of LCPECs

The LCPECs were prepared by combining PDADMAC and an LC polyanion synthesized via a one-pot two-step polymerization strategy designed to precisely control the charge density on the polymer (Figure 1a). In the first step, anionic oligomers bearing acrylate end groups were prepared by Michael addition between an excess of the LC monomer A and the ionic monomer B_1_ (Figure 1b). Subsequently, the remaining acrylates underwent thiol–ene reaction with a neutral dithiol monomer B_2_ to yield the LC polyanion. Monomer A was chosen as it is one of the most common LC diacrylate building blocks able to construct LCEs through thiol–ene or Michael addition reactions.^32,36,37^ Monomer B_1_ (taurine) was used as it is the simplest molecule that both carries a negative sulfonate charge and a primary amine that can undergo two Michael additions with acrylates to grow the backbone of the polyanion by a step-growth approach. Finally, monomer B_2_ is a flexible dithiol spacer also often used to prepare main-chain LCEs, with the thiol groups undergoing a thiol–ene click reaction with the acrylates.^32,36,37^ To ensure high conversion and molecular weight, the total molar ratio between monomer A and the combined monomers (B_1_ + B_2_) was strictly maintained at 1:1, while the ratio of B_1_ to B_2_ was varied to produce LC polyanions with varying charge densities (S_1_–S_3_) (Figure 1c).^38^ Additionally, a noncharged polymer consisting solely of monomers A and B_2_ (S_4_) was synthesized as a neutral reference for comparative studies. The complete disappearance of acrylate proton peaks (5.5–6.5 ppm) in the ^1^H NMR spectra confirmed high polymerization conversion for samples S_2_, S_3_, and S_4_, while S_1_ showed residual acrylate signals (conversion of monomer A, p_A_ = 91%), likely due to steric hindrance limiting the addition of a second acrylate to the amine (Figures S1–S4).^39^ For samples S_2_ and S_3_, a significant enrichment of the LC polyanion in monomer B_2_ was observed compared to the targeted values (A:B_1_:B_2_ = 1:0.26:0.74 for S_2_ and A:B_1_:B_2_ = 1:0.16:0.84 for S_3_). Nevertheless, the targeted trend of decreased charge density along the LC polyanion backbone from S_2_ to S_3_ was maintained. In addition, while we were not able to determine experimentally the molecular weight of the LC polymers, the complete conversion of monomer A for S_2_, S_3_, and S_4_ through this step-growth polymerization process is expected to lead to the formation of chains with relatively long molecular weights (>10^5^ g/mol). Oppositely, the LC polyanion used for S_1_ is expected to exhibit significantly lower molecular weight because of the incomplete conversion of monomer A. In the final step, PDADMAC aqueous solution was added to LC polyanion solution targeting a 1:1 positive-to-negative charge ratio between polyelectrolytes to form the corresponding LCPECs (Figure 1d). The resulting LCPECs were equilibrated overnight at room temperature, thoroughly washed with deionized water to remove residual salt and solvent, and hot-pressed prior to characterization. This synthetic strategy simplifies the experimental procedures and avoids the need for intermediate purification, while offering gradual tuning of the charge density through a single compositional variable (B_1_:B_2_ ratio). Moreover, the use of monomer A as a mesogenic building block promotes liquid crystallinity, providing an additional functionality in the material. At this stage, it is important to recognize that a substantial mismatch in charge density is expected between our LC polyanions and PDADMAC.^40^ This charge asymmetry is anticipated to affect the extent of intrinsic polyelectrolyte–polyelectrolyte electrostatic pairing, while promoting the retention of extrinsic polyelectrolyte–counterion pairs. The resulting presence of extrinsic pairs may maintain relatively high chain mobility and thereby provide a means to tune the viscoelastic properties of the resulting LCPECs.^8,9,41–43^ The combination of synthetic accessibility and compositional tunability makes this approach a versatile platform for exploring structure–property relationships in water-free and salt-free PECs.

**Figure 1:**
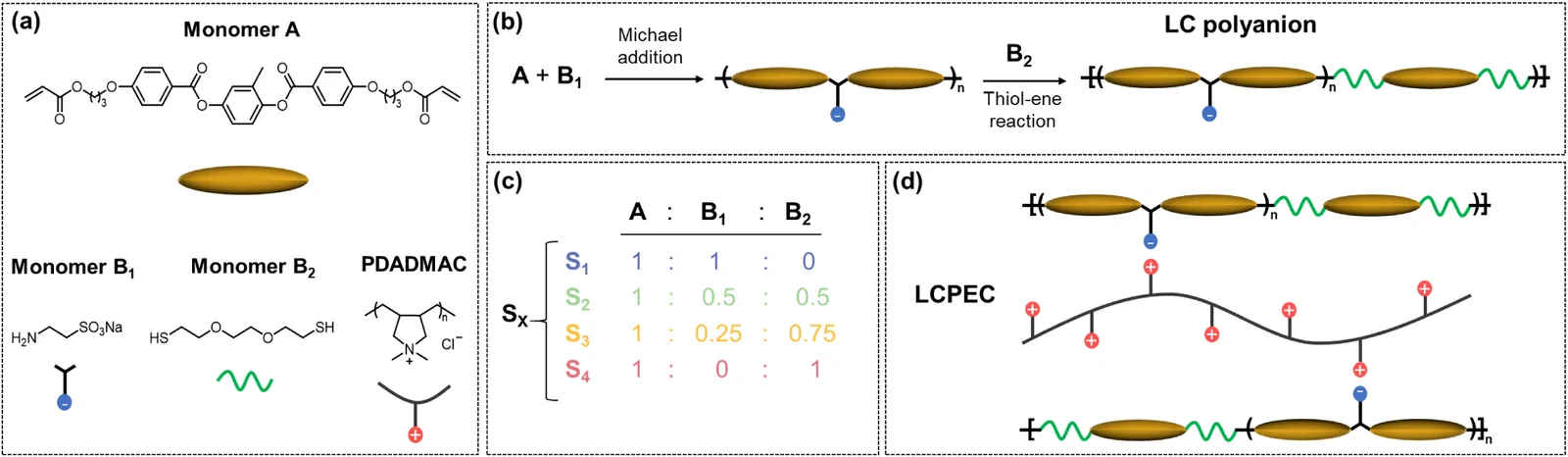
(a) Structure of the building blocks used in this work. (b) Schematic representation of the synthesis of the LC polyanion and (c) relative proportion of monomers A, B_1_, and B_2_. The LCPECs with varying molar ratios between monomers A:B_1_:B_2_ reported in the table are named from S_1_ to S_4_. (d) Schematic representation of the formation of the LCPECs by complexation of the LC polyanion with PDADMAC.

### 3.2 Thermal and Microstructural Characterization

The thermal properties of the LCPECs were first investigated with TGA, which demonstrated the absence of residual solvent and the good thermal stability of LCPECs with no significant degradation until approximately 300 °C for all samples (Figure S5). DSC further revealed glass transition temperatures (*T*_g_) around 58 °C for S_1_ with the highest charge density and no flexible neutral monomer B_2_, while the introduction of B_2_ led to a significant decrease in *T*_g_ to between 0 °C and −10 °C for other samples (Figure 2a). This reduction can be attributed to the increased chain flexibility imparted by the aliphatic segments of B_2_ and the reduced ionic interactions arising from the reduced density of ionic interactions that typically restrict chain mobility by acting as physical cross-links. The noncharged reference polymer (S_4_) exhibited an endothermic peak at 64 °C, corresponding to the LC–isotropic phase transition, as confirmed by POM images where birefringence completely disappeared above this temperature (Figure S6). For the LCPECs S_1_ to S_3_, this transition became broader and less pronounced in DSC measurements. This suppression is attributed to the ionic cross-links, which restrict the cooperative molecular rearrangements necessary for a well-defined phase transition, consistent with previous reports on covalently cross-linked liquid crystalline polymers.^44^

**Figure 2:**
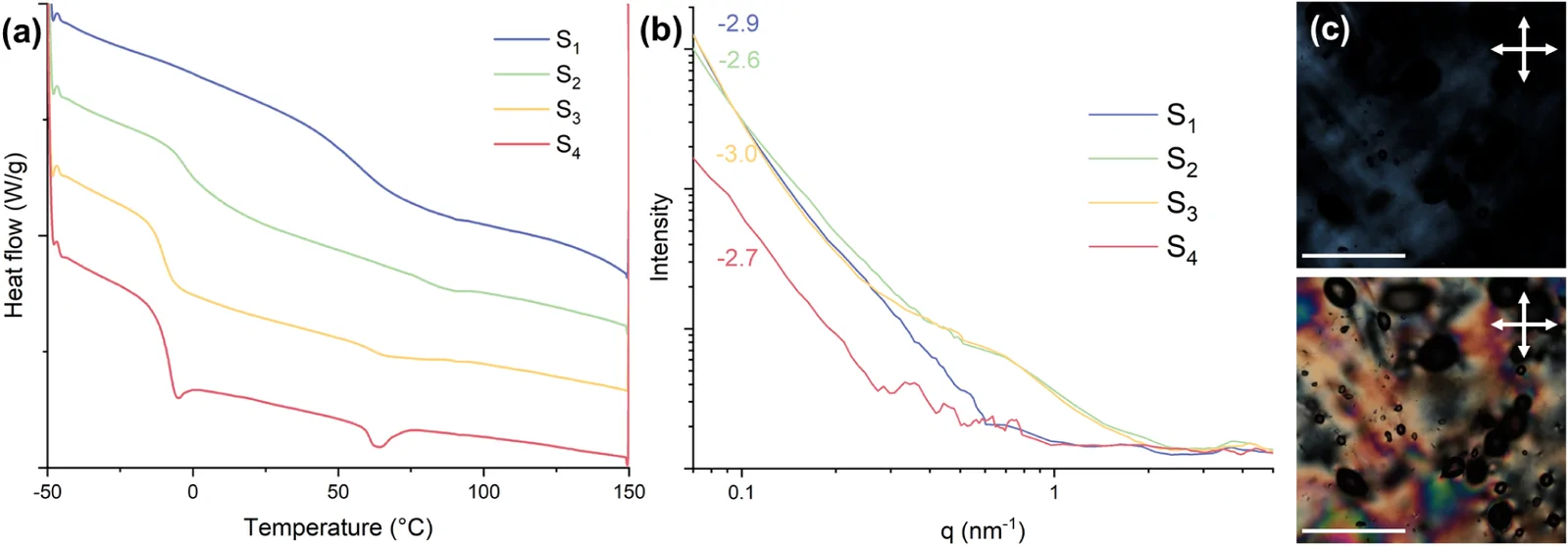
(a) DSC curves (exo up) and (b) SAXS 1D profiles of the LCPECs acquired at 20 °C. (c) POM images of S_3_ taken at 90 °C (top) and 50 °C (bottom). The scale bar is 0.5 mm.

Small-angle X-ray scattering (SAXS) provided insights into the mesoscale organization of the LCPECs (Figure 2b). In the low-*q* region (<0.3 nm^–1^), all samples exhibited power-law decay behavior of *I*(*q*) = *Aq* ^–α^, with α ranging from −2.7 to −3, indicating that the LCPECs do not exhibit a simple homogeneous network structure but rather a nonuniform fractal-like structure with weak segregation.^45,46^ These large-scale inhomogeneous architectures are commonly reported for polymer networks including PECs. The phase separation into PEC granules that may not fully coalesce into a homogeneous polymer network due to possible chains kinetic trapping often leads to an additional contribution to the detected inhomogeneities.^47–49^ Interestingly, S_2_ and S_3_ displayed a broad scattering feature located at approximately *q*_max_ = 0.62 nm^–1^, corresponding to a real-space distance of 2π/*q_max_* ∼9 nm. This broad signal is absent in S_1_ and S_4_, demonstrating that it arises from local aggregation occurring in the polyelectrolyte complex when both the ionic B_1_ and the flexible B_2_ monomers are present. The absence of scattering signals around 4 nm, which is the characteristic layer spacing in smectic LC polymers using similar mesogens, suggests that these LCPECs likely adopt a nematic-like phase, in which the mesogenic units maintain orientational order without positional periodicity.^32^ To confirm this, the LC–isotropic transition in these samples was clearly evidenced by POM observations, which showed the gradual appearance of birefringence upon cooling from 90 to 50 °C (Figure 2c). Wide-angle X-ray scattering (WAXS) was employed to characterize the local molecular ordering within the LCPECs. All samples exhibited a peak at around *q* = 14.8 nm^–1^ (d = 0.42 nm), attributed to the intermolecular spacing between adjacent mesogenic groups (Figure S7a).^32^

### 3.3 Mechanical Properties

The mechanical properties of LCPECs with varying charge densities were systematically investigated through rheological temperature sweeps from 140 to 20 °C to understand the relaxation mechanism and structure–property relationships (Figure 3a). Both storage (G′) and loss (G″) moduli increased with charge density, which suggested that the electrostatic interactions between the LC polyanion and PDADMAC effectively restricted chain mobility and stiffened the polymer network. The crossover temperature, where G″ overtook G′, evidencing the onset of viscous flow, shifted from 93 °C (S_4_) to 126 °C (S_3_) and further out of the investigation temperature range with increasing charge density. This progressive shift also demonstrates that the electrostatic cross-links impose an increasingly severe constraint on chain relaxation and thereby delay chain relaxation processes as the charge density increases. An additional feature was observed in S_4_, which exhibited a distinctive dip in both moduli corresponding to the LC–isotropic transition observed in DSC. This rheological signature of phase transition was absent in the LCPECs, consistent with our earlier DSC observations where the ionic cross-links suppressed the sharp thermal transitions characteristic of conventional liquid crystalline polymers.

**Figure 3:**
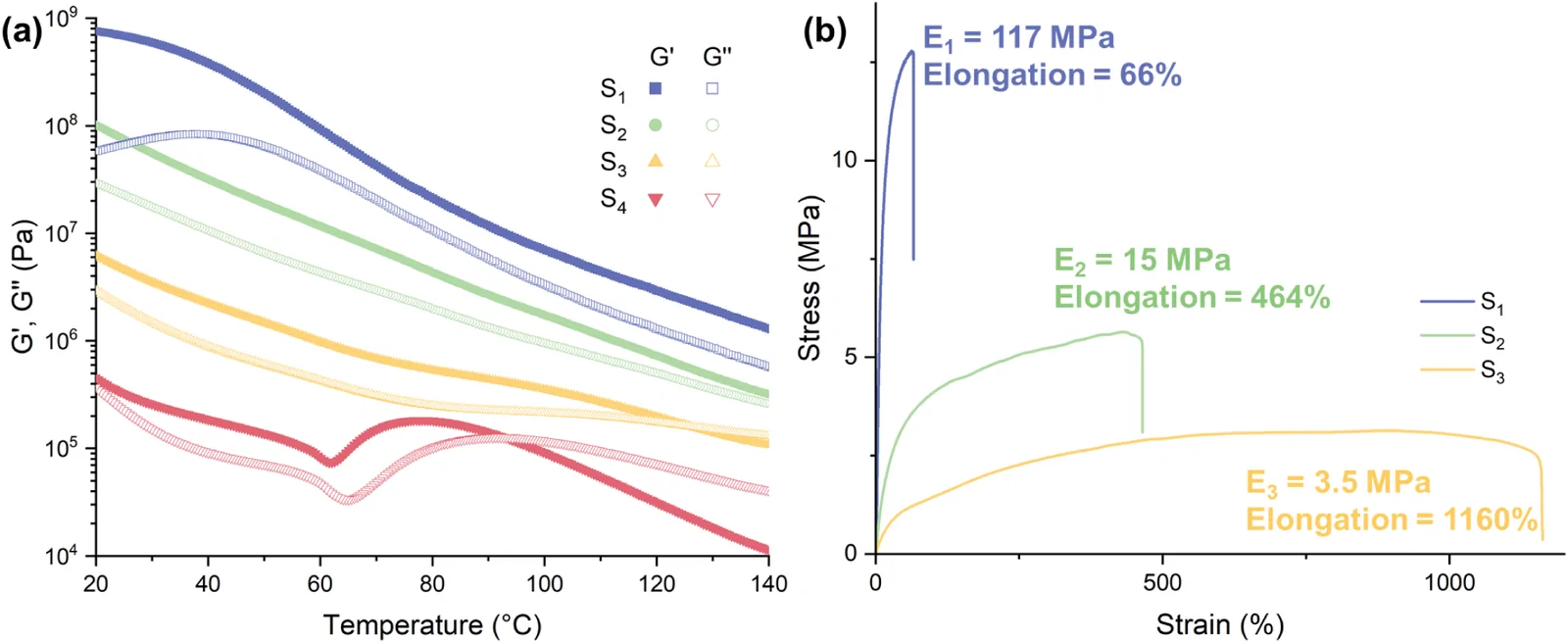
(a) Temperature sweep of the LCPECs from 140 to 20 °C at 10 rad/s. (b) Tensile testing results of the LCPECs at 20 °C under 50% relative humidity.

Tensile testing performed at room temperature under RH = 50% relative humidity (Figure 3b) provided critical insights into the dependence of the mechanical response of the LCPECs on polymer composition and the feasibility of mechanical alignment. S_1_ (LC polyanion with the highest charge density) exhibited the highest Young’s modulus E_1_ = 117 MPa but a limited fracture elongation of only 66%, characteristic of a stiff yet brittle material. As the charge density decreased, a drastic brittle-to-ductile transition was observed, with S_2_ and S_3_ (LC polyanion with the lowest charge density) displaying a substantially reduced Young’s modulus of, respectively, E_2_ = 15 MPa and E_3_ = 3.5 MPa but an extraordinarily high fracture elongation of, respectively, 464% and 1160%. Notably, the stretchability far exceeds the maximum stretchability reported for other water-free and salt-free PECs, where values up to 16% were obtained in the presence of an ionic liquid plasticizer.^16,23^ It is important to notice that identical mechanical properties were obtained for the LCPEC samples equilibrated overnight at room temperature either under vacuum (RH = 0%) or in a humid atmosphere (RH = 80%), excluding significant plasticization due to water uptake (Figure S8). In addition, despite S_1_ containing possibly an LC polyanion with significantly smaller molecular weight that could lead to softening of the LCPEC, it actually exhibited the highest stiffness and brittleness of all the LCPEC samples. This exceptional enhancement in stretchability can then be attributed to the complementary roles of two structural components: the ionic interactions maintain mechanical integrity and elastic recovery, while the neutral flexible segments provide chain mobility and extensibility. The noncharged reference S_4_ was too soft for hot-pressing and conventional tensile testing, highlighting the critical role of ionic interactions in providing mechanical integrity to these materials. Based on these findings, S_3_ was selected for alignment experiments, as it offered the optimal combination of strength and stretchability required for effective mesogen alignment via mechanical stretching without premature fracture.

### 3.4 Mesogen Alignment, Thermal Actuation, and Melt Electrowriting

To explore the feasibility of inducing liquid crystalline alignment through mechanical stretching, dog-bone-shaped specimens of S_3_ were subjected to uniaxial stretching to 500% strain at room temperature and a heating–cooling cycle was performed when strained. Upon cooling, a spontaneous elongation was observed, consistent with the formation of an aligned LC phase with the molecular director parallel to stretching. An additional stress-free heating–cooling cycle between 90 °C and room temperature was performed to remove residual mechanical stress while preserving the programmed alignment. POM images revealed a clear difference in birefringence intensity between a horizontally displaced and a 45°-tilted sample, indicating a uniaxial molecular alignment (Figure 4a). This optical anisotropy was then quantitatively confirmed by 2D WAXS measurements, in which the scattering peak previously identified as the intermolecular spacing between adjacent mesogenic units and located at 14.8 nm^–1^ (0.42 nm) transformed from an isotropic ring pattern in the unstretched state (Figure S7b) to a distinct pair of symmetric arcs perpendicular to the stretching direction (Figure 4b). This anisotropic pattern clearly demonstrated that the mesogens aligned parallel to the applied mechanical force. Azimuthal integration of the scattering intensity yielded an order parameter of 0.34 (Figure 4c), a value comparable to those reported for lightly cross-linked liquid crystalline elastomers prepared via similar procedures.^31^

**Figure 4:**
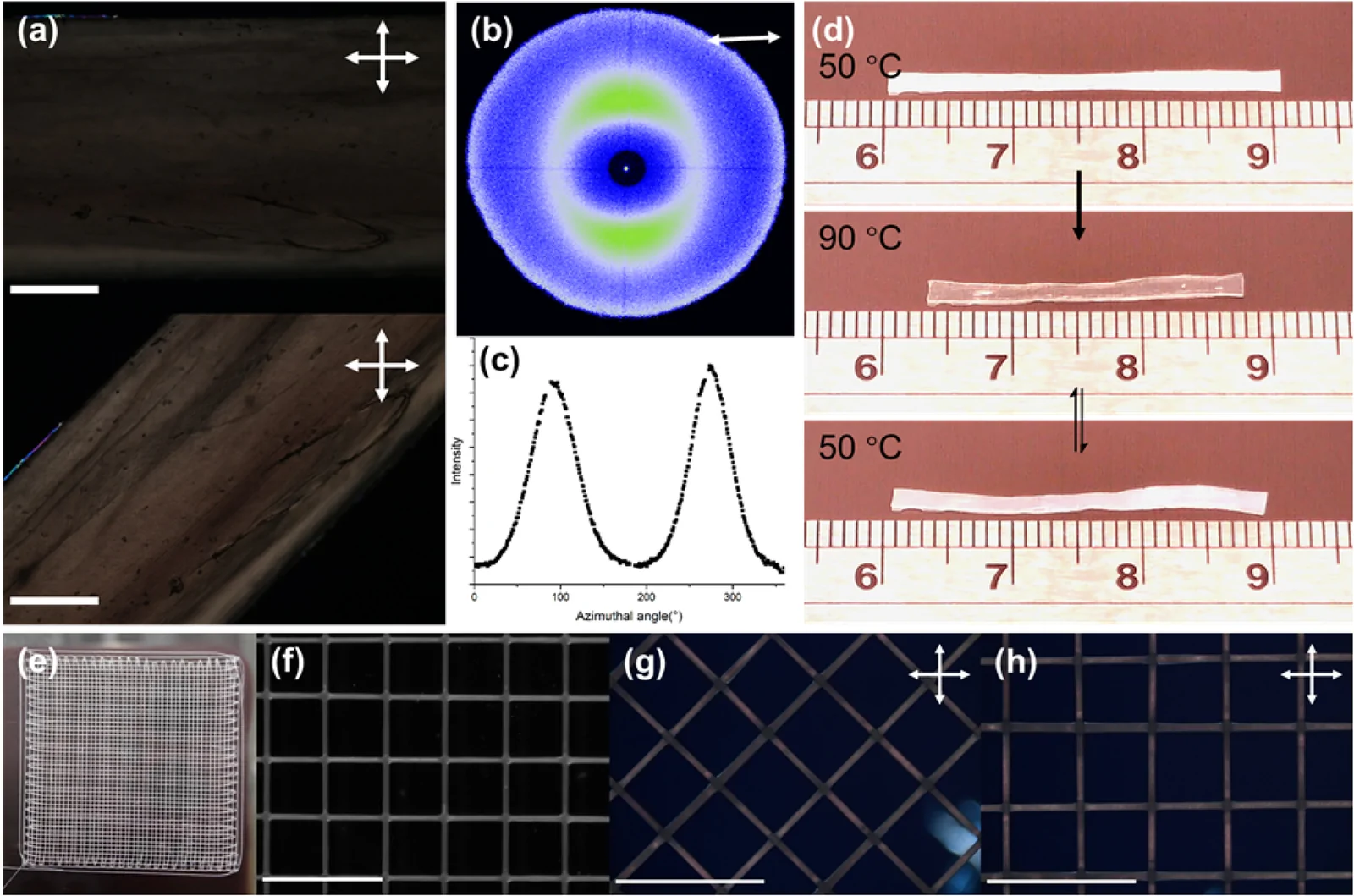
(a) POM images of the aligned S_3_ specimen tilted by 45° between each picture. Scale bar = 0.5 mm. (b) 2D WAXS pattern and the corresponding (c) 1D WAXS profile of the aligned specimen. The stretching direction is indicated by the top-right arrow. (d) Thermal actuation performed on an aligned specimen between 50 and 90 °C. (e) Melt electrowritten mesh structure of S_3_. (f) Corresponding optical microscopy picture without cross-polarizers. (g) and (h) POM images of the printed structure tilted by 45°. Scale bar = 1 mm.

The actuation behavior of the aligned S_3_ specimen was investigated by monitoring dimensional changes during thermal cycling between 50 and 90 °C, a temperature range spanning the LC–isotropic transition (Figure 4d and Movie S1). A programmed specimen with an initial length of 3 cm was placed on a hot plate preheated to 50 °C and subsequently heated to 90 °C. Upon heating above the LC–isotropic transition temperature, the specimen underwent a contraction to 2.4 cm (20% reduction in length) accompanied by a visible transition from translucent to transparent, indicating the disruption of the aligned liquid crystalline phase and the transition to an isotropic state. Then, upon cooling to 50 °C, the specimen elongated to 2.9 cm and simultaneously returned to its initial translucent appearance, indicating the recovery of nematic mesogenic alignment. Since the specimen slightly buckled in the cooling process, it was reasonable to conclude that the actuation was almost fully reversible. This reversible thermal actuation behavior, never before observed in PECs, demonstrates that the electrostatic interactions between polymer chains effectively act as dynamic cross-links that preserve the programmed molecular alignment while allowing sufficient chain mobility for the conformational changes necessary for actuation.

Finally, to demonstrate the advanced processability of our LCPEC system and explore its potential for precision manufacturing, we employed melt electrowriting (MEW) to fabricate well-defined mesh structures (Figure 4e). The LCPEC S_3_ was printed at 150 °C and a three-layer square-mesh scaffold was successfully prepared with strands exhibiting an average diameter of 60 ± 6 μm. Optical microscopy revealed smooth, defect-free fibers with well-defined intersections, confirming excellent material homogeneity and process stability (Figure 4f). The printed meshes displayed birefringence under POM, indicating that the mesogens reformed their nematic ordering upon cooling from the printing temperature (Figure 4g and 4h). However, no macroscopic molecular alignment was discernible when the structure was tilted by 45° under cross-polarizers even though the material experienced high shear rates during the electrowriting process. This absence of shear-induced alignment is likely due to the rapid relaxation of the material once extruded, for which the cooling rate is not sufficiently fast to kinetically trap the alignment before the mesogens relax and lose the alignment, leaving a macroscopically isotropic state after printing.

## 4 Conclusion

In summary, we have developed a general strategy for overcoming the intrinsic brittleness of salt-free and water-free LCPEC systems while simultaneously introducing stimulus-responsiveness property. By tuning the ratio of ionic to neutral monomers and employing a one-pot two-step polymerization, a continuous control over the ionic cross-link density of the LCPECs was achieved. A nonuniform fractal-like structure and nanoscale phase separation (∼9 nm) between the ionic cross-link domains and neutral segments were revealed together with a nematic ordering of the mesogens. The ionic interactions serve as physical cross-links that stiffen the material and slow down chain relaxation dynamics, while the neutral flexible segments provide chain mobility necessary for stretchability, leading to PECs whose mechanical toughness far surpasses those of existing water- and salt-free PECs. Mechanical stretching induced mesogen alignment in the specimen, which in turn enabled reversible thermally driven actuation with 20% dimensional changes. Furthermore, we successfully employed melt electrowriting to fabricate precise mesh structures, highlighting the material’s potential for advanced manufacturing applications. This work introduces a novel PEC system that eliminates the inherent brittleness of traditional PECs while introducing programmable shape-changing capabilities and enhanced processability. The proposed molecular design allows, in principle, for the combination of any LC diacrylate, amine-functionalized anionic, and multifunctional thiol monomers to produce LC polyanions capable of forming LCPEC actuators with tunable mechanical properties. The mechanical properties are mostly dependent on the charge density and cross-linking density, while the LC ordering is dependent on the LC monomer. For instance, while we report a nematic phase, other LC phases can be obtained with different mesogen monomers. Our LCPEC system establishes a promising platform for applications in soft robotics, smart textiles, and biomedical devices.

## Supplementary Material





## Notes

Movie of the thermal actuation for sample S_3_ (MP4)
Additional experimental details, materials, and methods; synthesis procedures and ^1^H NMR spectra for all compounds; and TGA, POM, and WAXS characterization (PDF)
