# Supplementary material for: Stretchable Water-Free Liquid Crystalline Polyelectrolyte Complexes
Source: ACS Appl Polym Mater. 2026 Aug 4;8(15):12440–8. doi: 10.1021/acsapm.6c01534 (PMC13488137; doi:10.1021/acsapm.6c01534)
Supplement: Supplementary file 1 [file ap6c01534_si_002.pdf]

Supporting information for

Stretchable Water-Free Liquid Crystalline Polyelectrolyte Complexes

Xiaohong Liu,[a,b*] Yuxuan Zhang,[c] Xixi Wu,[a,d] Rui Li,[a] Daniele Parisi,[e] Giuseppe Portale,[c] Marleen Kamperman[a,*], Julien Es Sayed[f,*]

[a] Polymer Science group, Zernike Institute for Advanced Materials, Faculty of Science and Engineering, University of Groningen, Nijenborgh 3, 9747 AG Groningen, the Netherlands.

[b] KINGFA Sci. & Tech. Co., Ltd., No. 33, Kefeng Road, Science Town, Guangzhou, China

[c] Physical Chemistry of Polymeric and Nanostructured Materials, Zernike Institute for Advanced Materials, Faculty of Science and Engineering, University of Groningen, Nijenborgh 3, 9747 AG Groningen, The Netherlands.

[d] School of Medicine & Nursing, Huzhou University, No. 759, Erhuan East Road, Huzhou, China.

[e] Department of Chemical Engineering, Engineering and Technology Institute Groningen, University of Groningen, Nijenborgh 3, 9747 AG Groningen, The Netherlands.

[f] Biotechnology Centre, The Silesian University of Technology, B. Krzywoustego 8, 44-100, Gliwice, Poland.

E-mails: j.s.es.sayed@rug.nl, marleen.kamperman@rug.nl

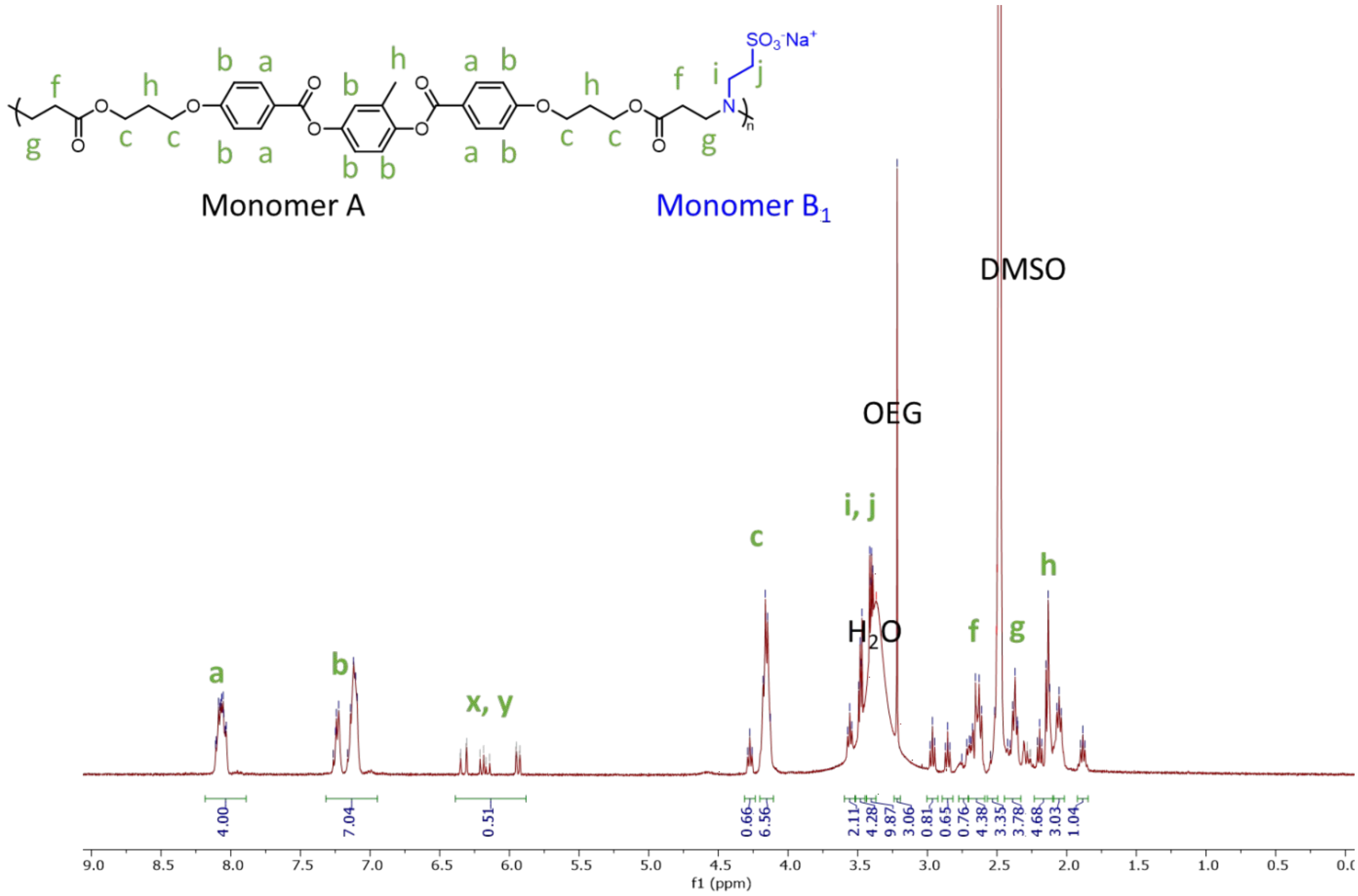

Figure S1. Chemical structure and ^{1}H NMR (DMSO-d_6) spectrum of the LC polyanion before the addition of PDADMAC for the preparation of the sample S_1. The peak named OEG is the residual diglyme used for the LC polymer synthesis. The peaks at 5.5 to 6.5 ppm corresponds to the unreacted acrylate groups of monomer A. The conversion (p_A) of monomer A is determined as follows: $p_A = 100 * [1 - Int (x,y) / 6] = 100 * [1 – 0.51/6] = 91.5\%$. The relative proportion of monomer A and B_1 in the LC polyanion is not possible to calculate from this spectrum due to the overlap of the water peak in DMSO-d_6 and the peaks i-j corresponding to the aliphatic protons of monomer B_1.

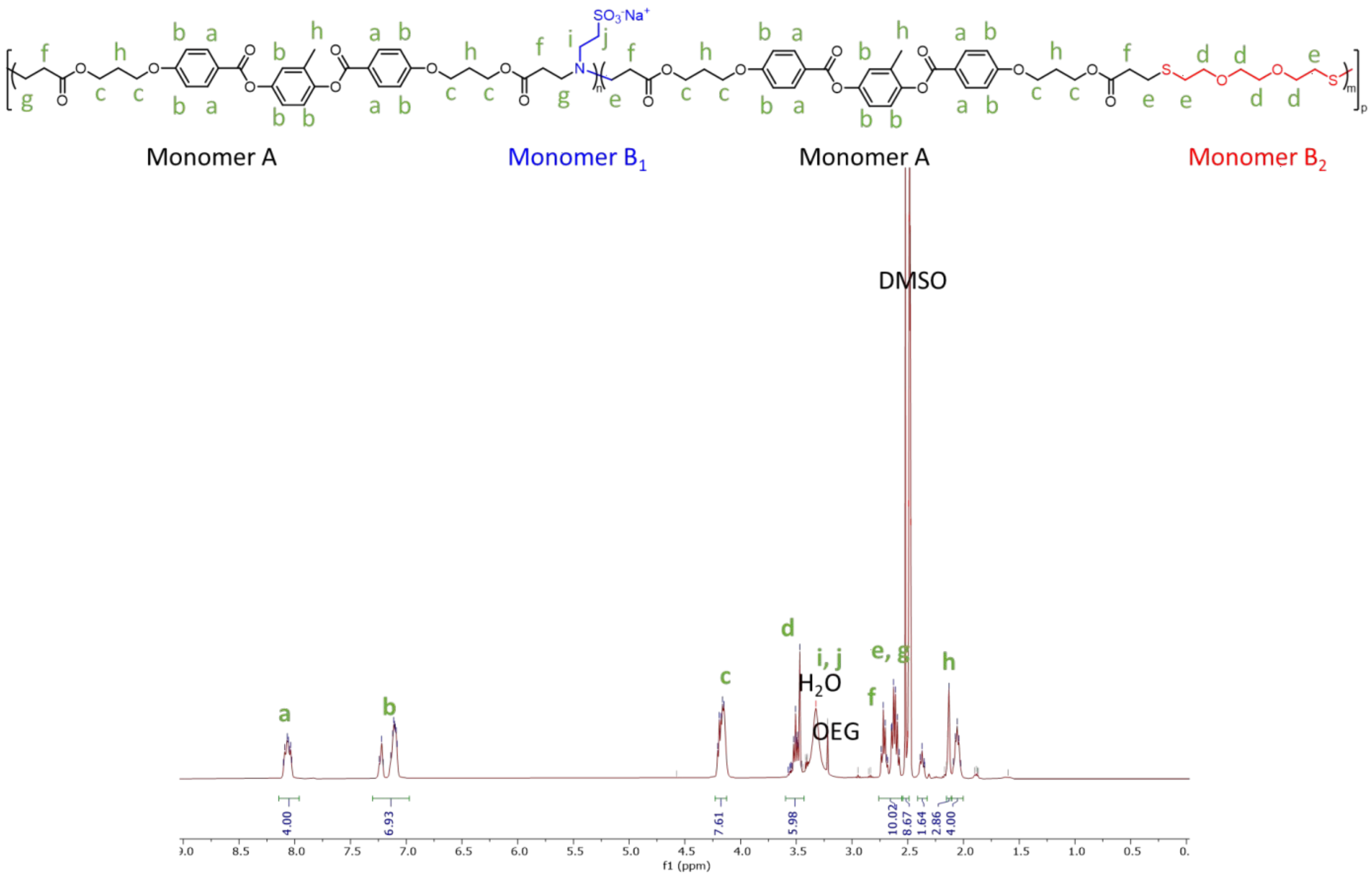

Figure S3. Chemical structure and ^{1}H NMR (DMSO-d$_6$) spectrum of the LC polyanion before the addition of PDADMAC for the preparation of the sample S_2. The peak named OEG is the residual diglyme used for the LC polymer synthesis. The absence of residual acrylate peaks confirms complete conversion of monomer A. The relative proportion of monomer A and B_2 in the LC polymer is calculated as follows: [A]:[B_2] = 2*Int (a) / Int (d) = 8/5.98 = 1:0.74 which deviates from the 1:0.5 value targeted but is line with the monomer incorporation trend we are targeting. As the peaks i-j corresponding to the aliphatic protons of monomer B_1 overlap with the water peak in DMSO-d$_6$ we are only able to perform indirect quantification of the relative content of monomer B_1 based on the full conversion of monomer A. Consequently, we infer a relative ratio [A]:[B_1] = 1 - [A]:[B_2] = 1:0.26.

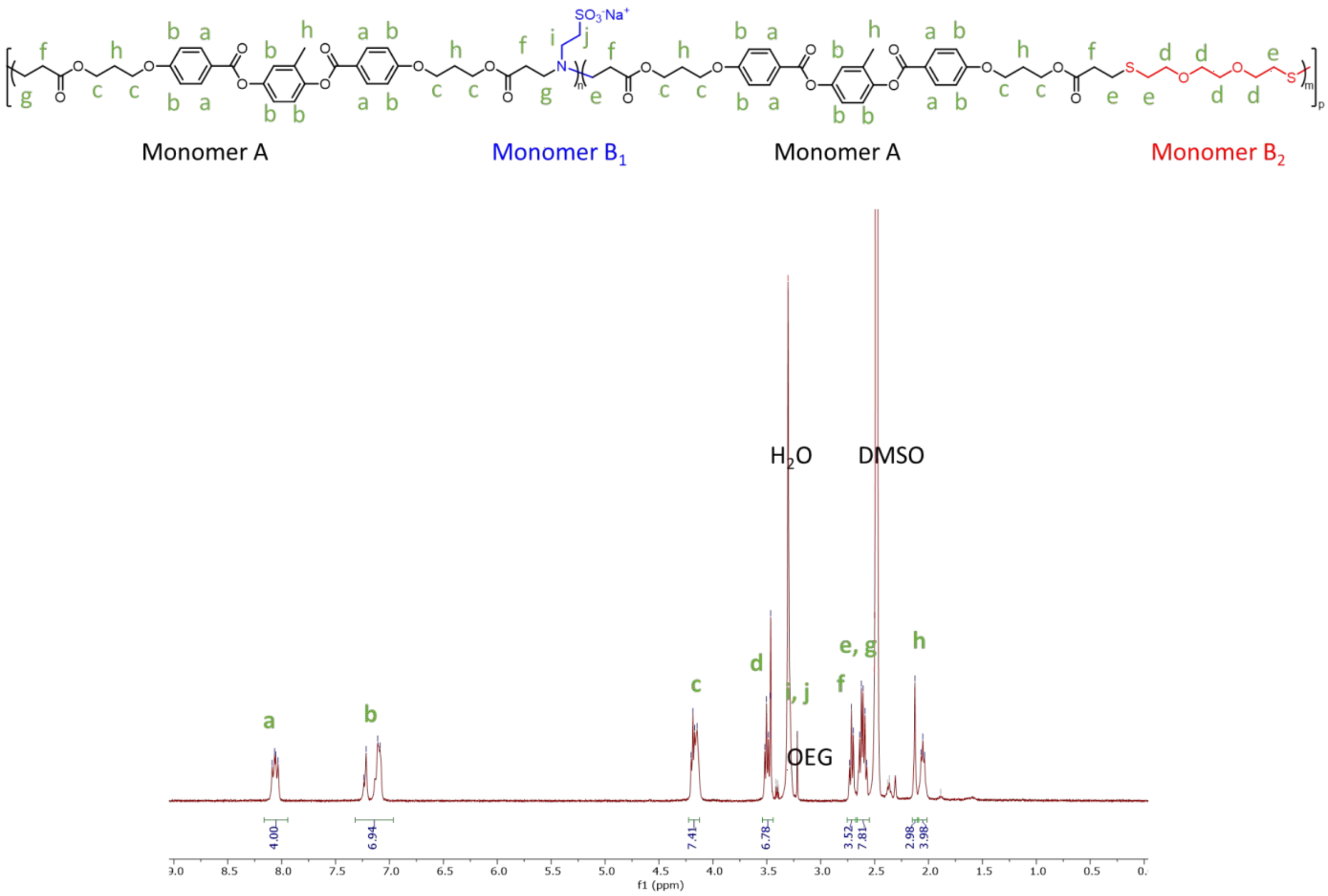

Figure S3. Chemical structure and ^{1}H NMR (DMSO-d_6) spectrum of the LC polyanion before the addition of PDADMAC for the preparation of the sample S_3. The peak named OEG is the residual diglyme used for the LC polymer synthesis. The absence of residual acrylate peaks confirms complete conversion of monomer A. The relative proportion of monomer A and B_2 in the LC polymer is calculated as follows: $[A]:[B_2] = 2*Int (a) / Int (d) = 8/6.78 = 1:0.84$ which deviates from the 1:0.5 value targeted but is line with the monomer incorporation trend we are targeting. As the peaks i-j corresponding to the aliphatic protons of monomer B_1 overlap with the water peak in DMSO-d_6 we are only able to perform indirect quantification of the relative content of monomer B_1 based on the full conversion of monomer A. Consequently, we infer a relative ratio $[A]:[B_1] = 1 - [A]:[B_2] = 1:0.16$.

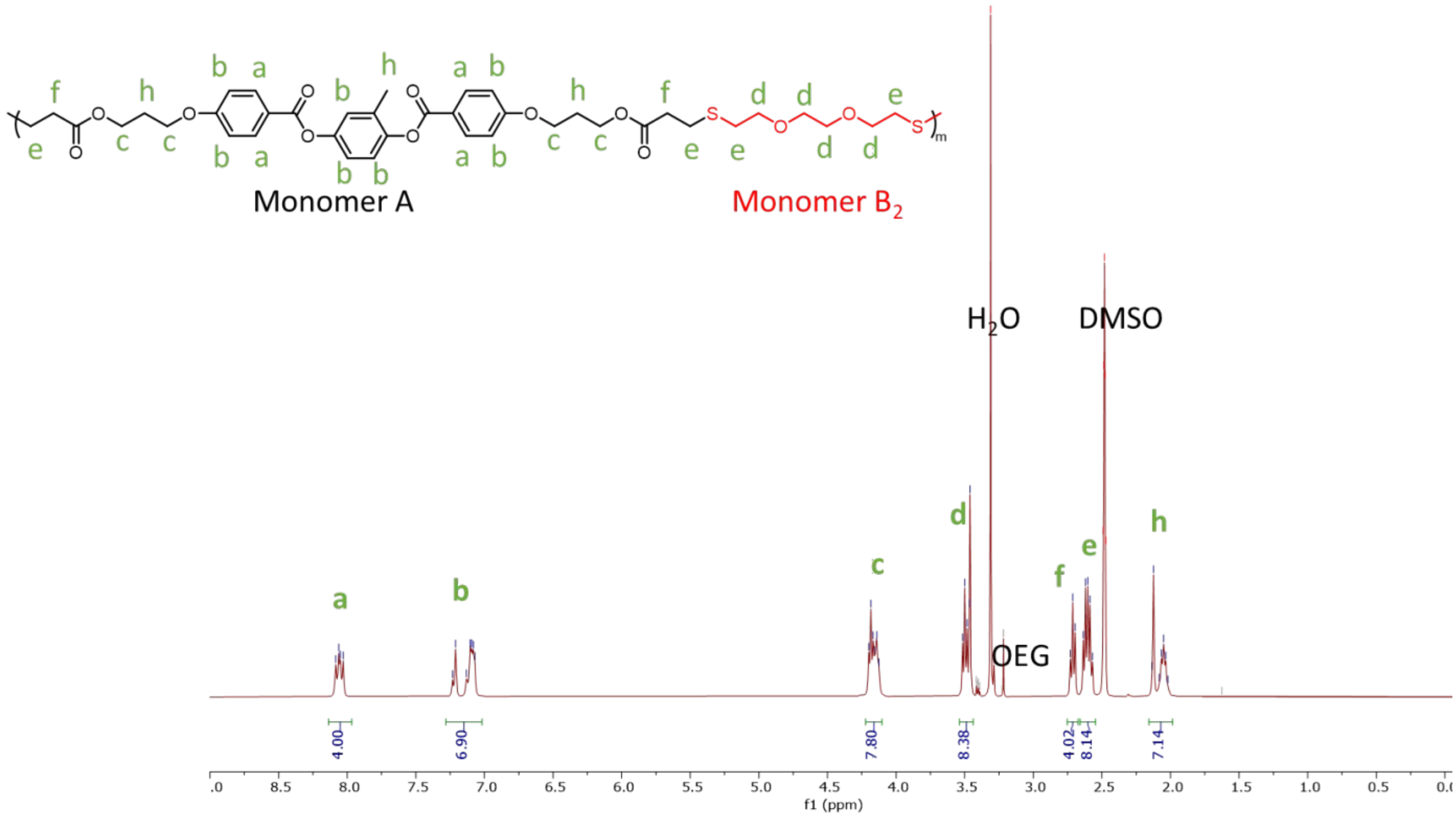

Figure S4. Chemical structure and ^{1}H NMR (DMSO-d_6) spectrum of the LC polyanion before the addition of PDADMAC for the preparation of the sample S_4. The peak named OEG is the residual diglyme used for the LC polymer synthesis. The absence of residual acrylate peaks confirms complete conversion of monomer A. The relative proportion of monomer A and B_2 in the LC polymer is calculated as follows: $[A]:[B_2] = 2*Int(a) / Int(d) = 8/8.38 = 0.96$ which is close to the 1:1 value targeted.

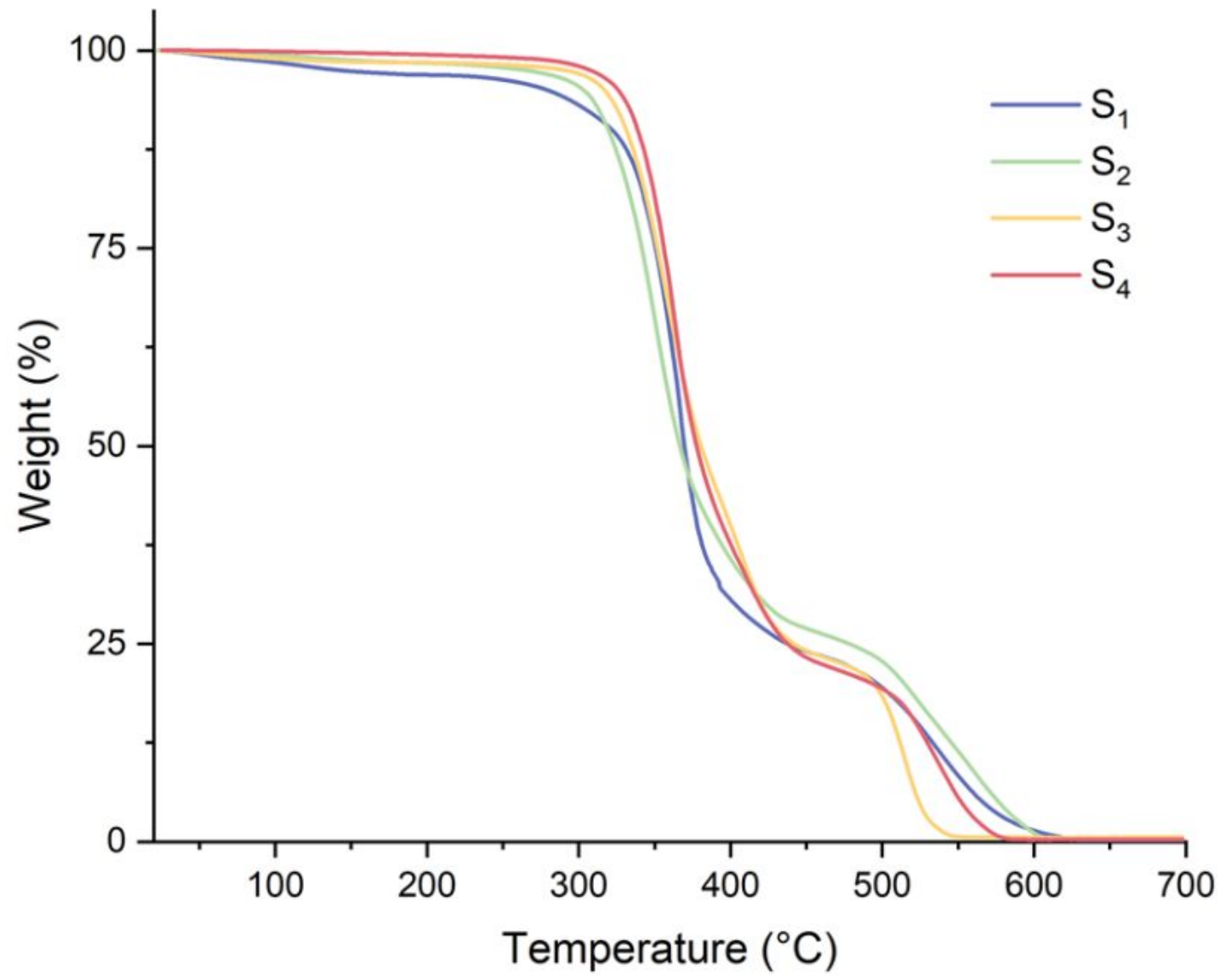

Figure S5. TGA curves of the LCPECs. No significant weight loss was observed until 300 °C.

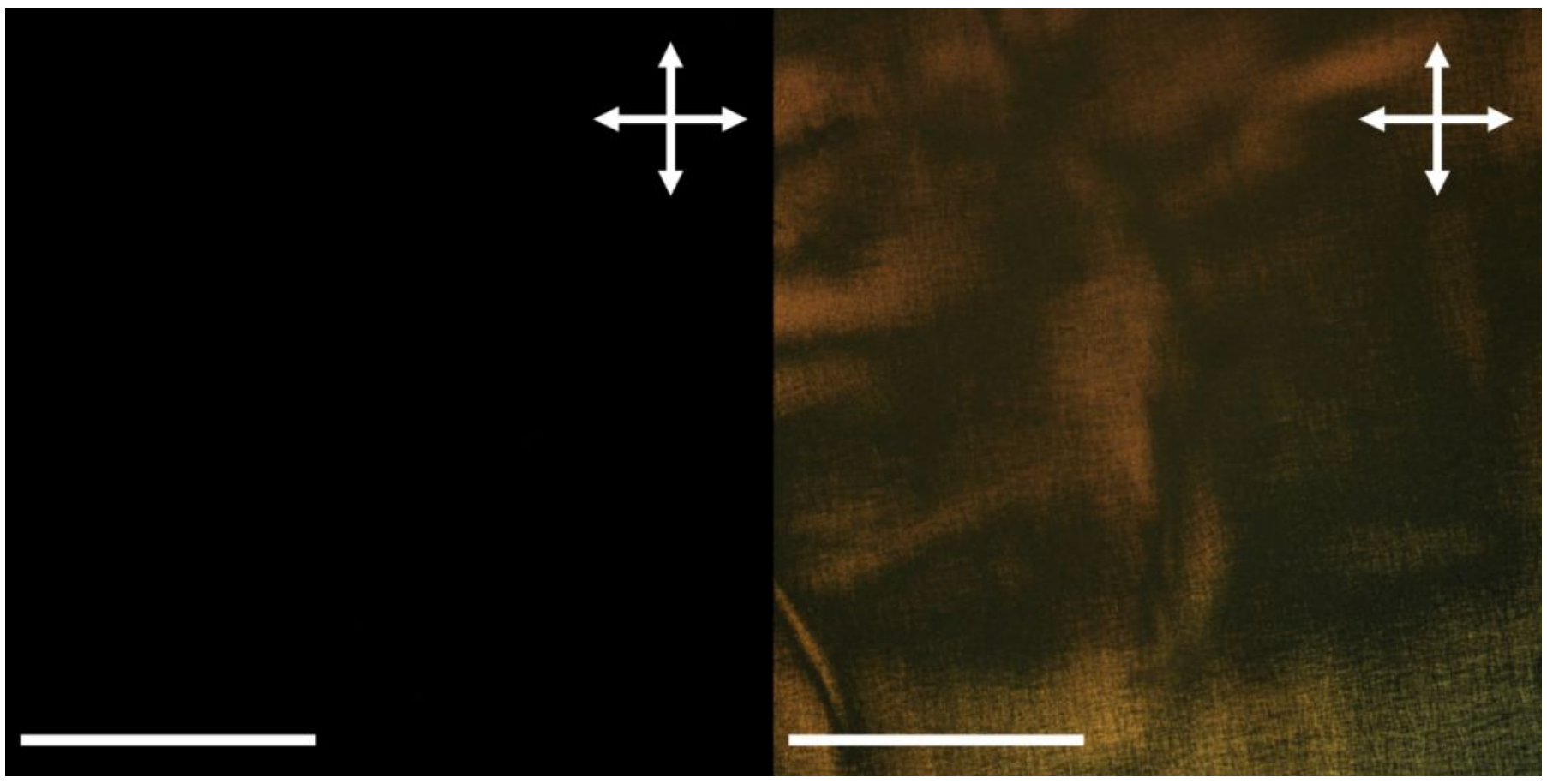

Figure S6. POM image of S_4 taken at 70 °C (left) and 60 °C (right). Scale bar = 1 mm.

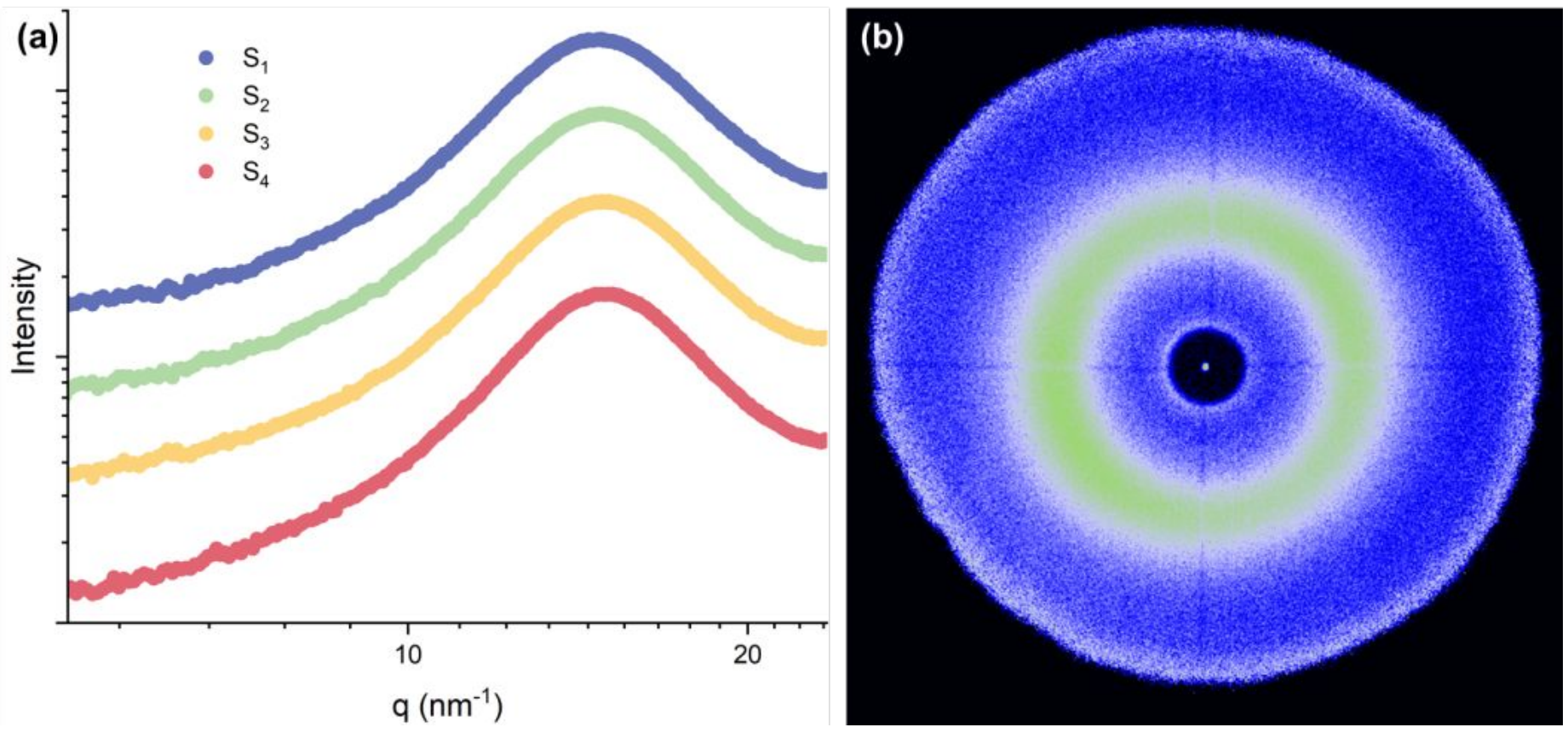

Figure S7. (a) 1D WAXS profile of the LCPECs. A peak at 14.8 nm^{-1} (0.42 nm) can be observed in all samples. **(b)** WAXS pattern of S_3.

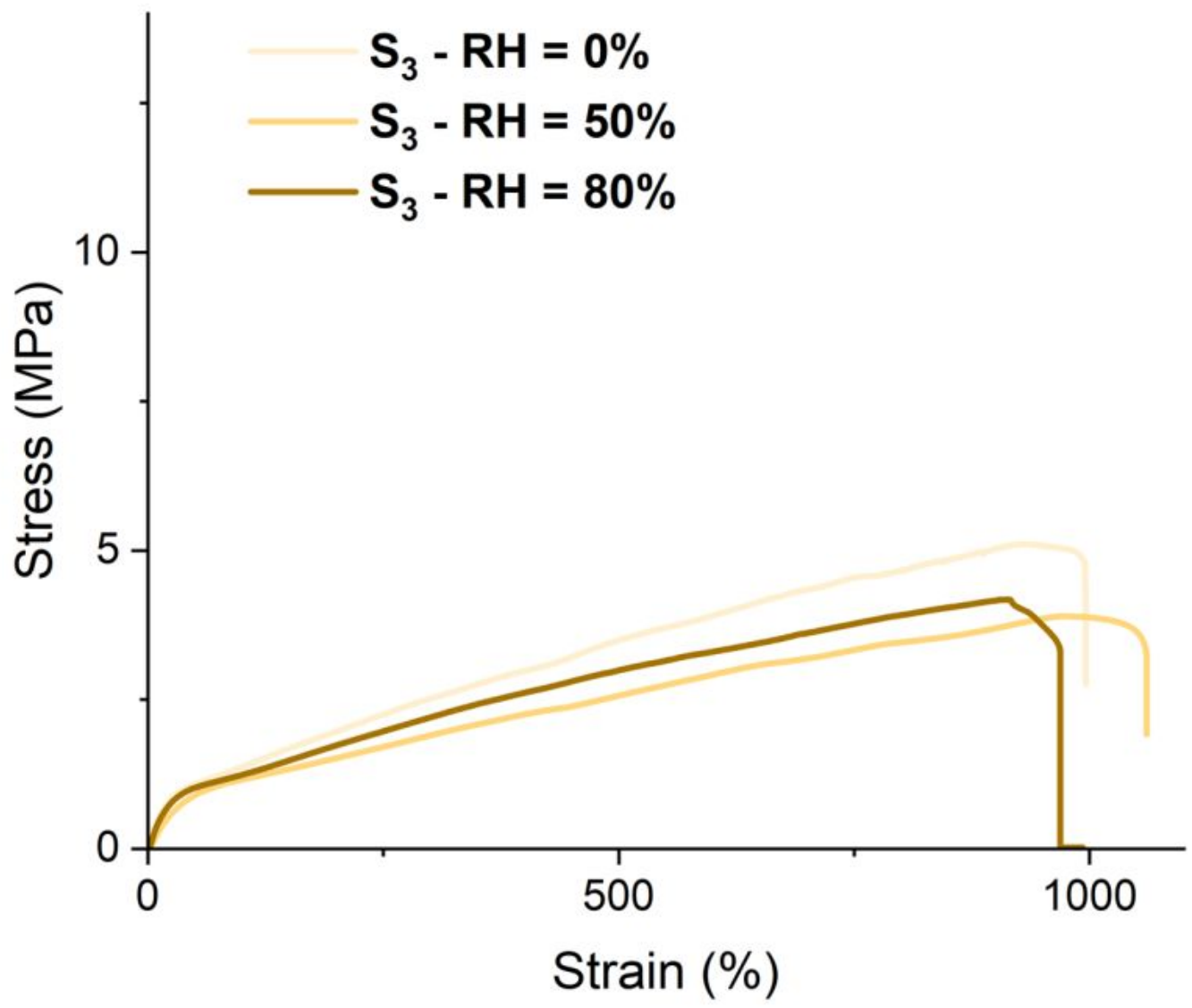

Figure S8. Tensile testing results of the LCPECs at 20 °C incubated overnight either in vacuum (3mbar, RH, relative humidity = 0%), in ambient air (RH = 50%) or in a humidity box (RH = 80%).

Movie S1. Thermal actuation experienced by the sample S_3.
